# Fundamentals or Icing on Top of the Cake? A Narrative Review of Recovery Strategies and Devices for Athletes

**DOI:** 10.3390/sports11110213

**Published:** 2023-11-03

**Authors:** Matthew Driller, Alana Leabeater

**Affiliations:** Sport, Performance, and Nutrition Research Group, School of Allied Health, Human Services and Sport, La Trobe University, Melbourne 3086, Australia; a.leabeater@latrobe.edu.au

**Keywords:** compression, ice baths, cold water immersion, sport performance, massage guns

## Abstract

The sport and athletic performance industry has seen a plethora of new recovery devices and technologies over recent years, and it has become somewhat difficult for athletes, coaches, and practitioners to navigate the efficacy of such devices or whether they are even required at all. With the increase in recovery devices and tools, it has also become commonplace for athletes to overlook more traditional, well-established recovery strategies. In this narrative review, we discuss recovery strategies in relation to the hierarchy of scientific evidence, classifying them based on the strength of the evidence, ranging from meta-analyses through to case studies and reports. We report that foam rolling, compression garments, cryotherapy, photobiomodulation, hydrotherapy, and active recovery have a high level of positive evidence for improved recovery outcomes, while sauna, recovery boots/sleeves, occlusion cuffs, and massage guns currently have a lower level of evidence and mixed results for their efficacy. Finally, we provide guidance for practitioners when deciding on recovery strategies to use with athletes during different phases of the season.

## 1. Introduction

The selection of recovery devices and technologies available for use by athletes has experienced substantial growth in recent years as athletes seek effective, time-efficient, and often portable methods to improve physical recovery after training and competition. New devices and strategies become rapidly popular and publicised on social media, while “recovery centres” charge a premium for access to a variety of devices, aimed at both athletes and the public alike. With this increased focus on “recovery” and these various devices and tools, it has become somewhat difficult for athletes, coaches, and practitioners to navigate the efficacy of such devices and determine if there is any risk of harm. It has also become commonplace for athletes to overlook more traditional, well-established recovery strategies like sleep and appropriate nutrition practices, contributing to a mismatch between what coaches rate as effective recovery strategies and what athletes rate as being the most effective [1].

Among the various assertions made by manufacturers of recovery devices, the primary objective is typically to alleviate muscle soreness, mitigate overall feelings of fatigue, and enhance an athlete’s capacity to tolerate their training workload. Some strategies or devices may concomitantly target other aspects of recovery that may aid physiological (e.g., increased blood flow and reduced metabolic waste), biomechanical (e.g., increased range of motion), neurological (e.g., reduced central nervous system fatigue), or psychological (e.g., increased mood, motivation, and readiness to train) factors, but essentially, a key component of most of these devices is to reduce the perception of soreness and increase feelings of recovery. At present, it is difficult to establish the validity of these claims, as the popularity of such devices precedes clear evidence from experimental research.

This narrative review will discuss the purpose of recovery strategies and the fundamentals of recovery (sleep, nutrition, and training periodisation), before categorising other recovery strategies by their current level of evidence. Additionally, this review will discuss the chronic use of recovery strategies and future considerations in this field of research.

## 2. The Purpose of Recovery

Put simply, training is the application of acute physical challenges to the body over time in order to maximise physiological capability [2]. Recovery, therefore, is the time outside of training where these improvements in physiological capability will actually transpire, and must be carefully balanced with training stress to optimise performance while avoiding maladaptation, injury, or illness [3]. Recovery modalities are implemented to act across various physiological, biomechanical, neurological, or psychological domains [4] (summarised in Table 1), though athletes primarily report reducing muscle soreness as the main purpose of implementing recovery strategies [1,5]. Muscle soreness results from mechanical disruption and inflammation of the muscle fibres, which causes the release of enzymes that sensitize the nerves within the muscle, producing pain when the muscle contracts or is stretched [6]. The inflammation and microtrauma that occur in the muscle cell are essential for the muscle tissue to strengthen and adapt, allowing them to produce force again without the same degree of damage occurring (the “repeated-bout effect”) [7]. While muscle soreness is unlikely to significantly impact athletic performance, excessive levels of muscle soreness may discourage athletes from engaging in further exercise or lead them to reduce intensity in subsequent training sessions [8]. Although, it should be noted that muscle soreness and delayed-onset muscle soreness (DOMS) in particular are usually associated with intense, unfamiliar, or eccentric-based exercise [8], rather than regular training. As such, it may be problematic to extrapolate the findings of recovery research conducted on lesser-trained participants to elite or highly-trained athletes who complete regular, familiar training [7].

Regular training is a necessary part of being an athlete, and without any recovery interventions, individuals will naturally recover at their own rate following exercise. However, the underlying theory behind utilising recovery interventions is that they have the potential to expedite this process [7]. Incorporating different recovery strategies might permit athletes to minimise the deleterious effects of muscle soreness and perceived fatigue, ultimately allowing for maintenance of subsequent training and competition performance. If a particular strategy alleviates muscle soreness, for example, this may in turn allow an athlete to train sooner, with greater quality and/or with a higher volume/intensity. This is based on the theory of supercompensation, whereby acute central and peripheral fatigue induced by an exercise bout can be restored by a period of recovery, allowing for physiological adaptations to take place and ultimately restoring or improving physiological capacity and athletic performance [3].

## 3. The Fundamentals of Recovery

**Figure 1:**
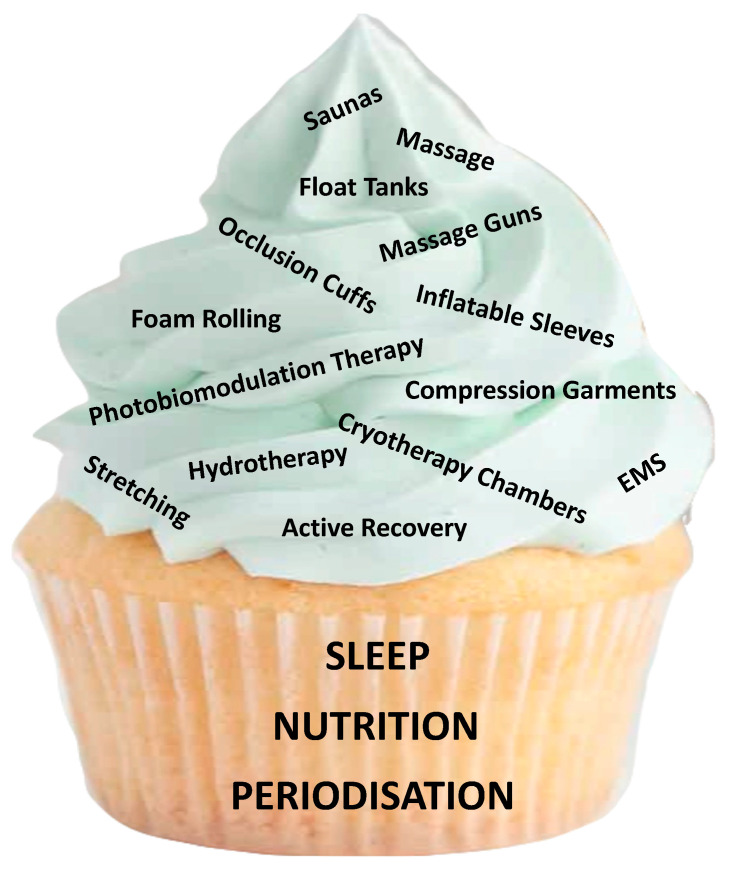
The fundamentals and examples of some “icing on top of the cake” strategies for recovery in athletes.

Athletes’ perceptions of recovery methods often do not align with current scientific evidence [5], and as such, athletes may overlook some of the more well-established methods of recovery in favour of new or novel recovery technologies. While research evidence is weak or sparse on many of these newer technologies like massage guns, occlusion cuffs, and recovery boots/sleeves, there is a greater level of empirical support for the fundamental recovery strategies of sleep, nutrition, and periodisation and their role in athletic recovery. These fundamentals (“the cake”; Figure 1) have a much greater overall contribution to athletic recovery and performance than any potential marginal improvements from added devices/tools (“the icing”) and must therefore be monitored and optimised before considering the implementation of further strategies or devices.

### 3.1. Sleep

Sleep is an essential process for the optimal maintenance of an athlete’s health and plays a critical role in the psychophysiological recovery of an athlete when many restorative bodily functions and processes take place. It is well recognised that sleep loss impairs numerous functions, including cognition, memory consolidation, immunity, and, importantly, athletic performance [9]. Despite this, elite athletes in the professional era are facing more intensive physical training loads, competition loads, and high levels of mental stress on a regular basis, resulting in several factors that could have an influence on sleep disturbances. Alongside this, various studies have identified that athletes may struggle to obtain the recommended 7–9 h of sleep per night due to different sport- (e.g., early morning training) and non-sport-related factors (e.g., study commitments), therefore compromising their recovery [9]. Yet, increasing nightly sleep duration can improve sprint time, reaction time, aerobic capacity, and body composition [10,11].

The importance that sleep has on the health, recovery, and performance of athletes is clearly highlighted by the rapid increase in research regarding sleep in athletic populations, with over 80% of the overall journal articles published in this field in the last decade [12]. Technological advancements in sleep measuring and monitoring have also advanced this field of research as wearable technologies approach the accuracy of research-grade actigraphy for detecting sleep and wake [13]. However, caution should be taken when using data from these wearables for other purposes, such as informing training, accurately identifying sleep phases, or diagnosing sleep disorders [14]. Simple sleep diaries or athlete-specific sleep questionnaires [15,16] can provide insight into areas for improvement, while sleep hygiene education may yield short-term improvements in sleep quality and duration in athletes [17,18,19].

### 3.2. Nutrition

Maintaining adequate dietary energy intake is paramount for athletes to promote energy availability for training and reduce the risk of injury and illness. Strategies for recovery nutrition will be sport- and sex-dependent [20], but the fundamentals are considered to be the following: (i) refuelling (replacing carbohydrates after exercise), (ii) rebuilding (intake of protein to aid in muscle repair and growth), and (iii) rehydrating (maintaining fluid intake, especially in the summer months) [21]. Meeting these primary needs and ensuring adequate nutritional intake (macro- and micro-nutrients) to meet overall energy requirements should be first and foremost in nutrition planning [22]. The use of certain nutritional supplements may also be encouraged to support training and competition demands, with guidance from previous reviews that have categorised the available evidence on these supplements [23,24,25].

### 3.3. Periodisation

Structured physical training is based upon the principles of progressive overload and adaptation, whereby an increasing training stimulus is applied over time to elicit physiological adaptations and improvements in performance [3]. Training load—comprised of training volume, intensity, and frequency—must be manipulated appropriately over microcycles (weeks), mesocycles (months), and macrocycles (seasons) to maintain or improve physiological capabilities [26]. Planning recovery within and across these cycles is necessary and should consider the characteristics of individual training sessions (e.g., metabolic demand), individual variations in the recovery process, and desired physical adaptations [26]. Time away from the sport/training environment, rest, social recovery, and downtime are all important factors that need to be considered in the overall periodised plan. Insufficient recovery will not only impact an athlete’s performance in subsequent training bouts but will also curtail the potential physiological adaptations from the initial training bout and thereby fail to meet the basic purpose of the training process [3].

The type and duration of recovery periods can be manipulated across a competitive season depending on the desired outcome; for example, withholding or limiting structured recovery interventions during a pre-season phase to promote physical adaptations to maximal training stress [27]. However, continuing to overload training volume (or any aspect thereof) while limiting recovery can place athletes at risk of non-functional over-reaching or overtraining [28]. The latter represents a chronic imbalance between training and recovery, manifesting primarily as a prolonged performance decrement, though other symptoms can be apparent [28]. This underlies the importance of athlete monitoring (including both objective and subjective measures) to capture an individual’s response to training load over time and inform acute adjustments to training as required [29].

## 4. Categorising Recovery Strategies

**Figure 2:**
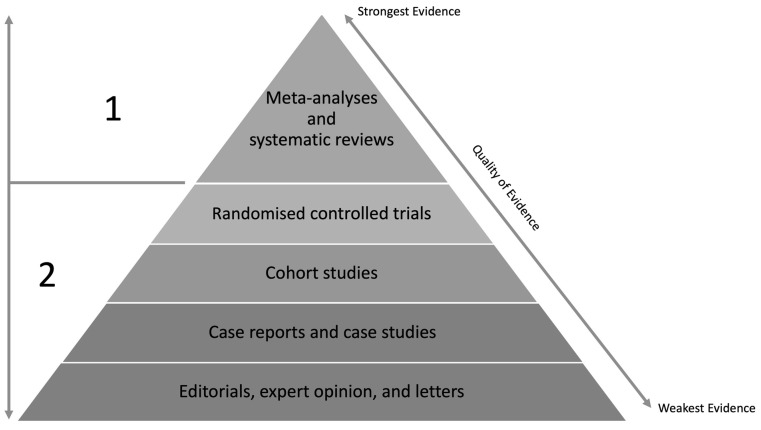
Hierarchy of scientific evidence to consider when evaluating different strategies and their efficacy.

To evaluate the efficacy and usefulness of various recovery strategies, the hierarchy of scientific evidence can be applied (Figure 2). This system of grading scientific evidence recognises that different study designs influence the reliability and validity of study results; however, it may overlook the overall feasibility of an intervention [30]. This is relevant when considering that some of the new or emerging recovery tools have only been evaluated in case studies or small-sized experimental trials (if at all), and therefore a meta-analysis or systematic review is not warranted. As such, this hierarchy for evaluating the quality of evidence can be seen as a guideline and a suitable starting point for assessing different recovery devices and strategies. The next section will discuss this hierarchy of evidence in two parts (as indicated by 1 and 2 in Figure 2 below) and briefly discuss the common recovery strategies/devices that fall under these categories. We acknowledge that there are other recovery strategies and devices that are used by athletes that are not covered in this review, and it is beyond the scope of this paper to discuss all recovery modalities; however, based on previous literature [1,31,32,33], we have selected some of the most popular strategies implemented by athletes.

### 4.1. High-Level of Evidence

The most rigorous level of evidence for a particular topic is often established through meta-analyses and systematic reviews, which summarise individual studies and the quality of these studies according to specific criteria. The following recovery strategies, which have been the subject of meta-analyses and/or systematic reviews, will be discussed in this section: foam rolling, compression garments, EMS, cryotherapy chambers, photobiomodulation, hydrotherapy, active recovery, and stretching. It is important to note that while certain strategies may have a high level of evidence (e.g., via meta-analyses and systematic reviews), that does not mean that the evidence is positive for that particular strategy.

#### 4.1.1. Foam Rolling

Foam rolling is a form of self-massage that is purported to enable myofascial release and improve flexibility and range of motion [34], and it has been the subject of a number of meta-analyses and reviews. Overall, it appears that foam rolling is an effective method of improving range of motion (ROM) following exercise [34], with the optimal “dose” being 90–120 seconds of rolling per muscle group [35]. As well, foam rolling appears to reduce DOMS and increase pressure-to-pain thresholds after exercise and therefore may optimise recovery from training [35]. While the ROM and DOMS benefits seem relatively consistent across studies and there appears to be no detrimental effect on recovery, the actual performance recovery benefits are less clear. Very few studies have shown that increased ROM or decreased DOMS translates to athletic performance (e.g., isokinetic strength, jumping, or agility) following foam rolling as a recovery strategy [36,37].

#### 4.1.2. Compression Garments

Compression garments (CGs) are a type of apparel used in both clinical and sports settings to apply mechanical pressure to body tissues and thereby improve blood flow. The practicality and portability of these garments lend themselves to their popularity, with reports that up to 71% of elite athletes sleep in CGs at least once per week [38]. In a meta-analysis of 23 studies, Brown et al. (2017) reported that CGs most effectively enhanced recovery from resistance exercise, particularly at time points > 24 h [39]. It was also suggested that compression garments might aid in next-day endurance performance, particularly cycling. More recent systematic reviews have reported that there is a trend for reduced lactate dehydrogenase and increased arterial blood flow during recovery with CGs, and the garments are consistently associated with decreases in perceived muscle soreness following fatiguing exercise [40,41]. As with foam rolling, there are little to no reported detriments to recovery or performance that have been reported with the use of CGs, and therefore, the potential benefits to physiological or psychological recovery are valuable.

#### 4.1.3. Electromyostimulation (EMS)

Electromyostimulation, sometimes referred to as electrical muscle stimulation (EMS) or neuromuscular electrical stimulation (NMES), involves the placement of surface electrodes on the skin overlying large muscle groups (e.g., quadriceps) with the aim of inducing muscle contractions to increase muscle blood flow and thus muscle metabolite removal. Largely inconsistent findings on this topic have been reported in systematic reviews, leaning towards the conclusion that the technique is mostly ineffective for recovery from exercise. In a 2014 review of 13 EMS studies, authors reported that only 1 study showed a performance recovery benefit and 4 showed a benefit to perceived muscle pain or exertion [42], with the remainder of studies showing no benefit of EMS compared to active or passive recovery methods. Similarly, a 2020 review of 11 studies reported inconsistent findings for both strength and endurance outcomes, though it is difficult to draw conclusions as there is a wide variety of devices implemented, protocols, electrode placement sites, and stimulation frequency/intensities [43]. These factors, and the potential for discomfort or pain with the use of these devices (depending on stimulation intensity), should be considered prior to their use.

#### 4.1.4. Cryotherapy Chambers

Whole-body cryotherapy (WBC) involves short exposures (generally between 2 and 4 min) to very cold air (around −100 °C or −150 °F) in a controlled room or chamber. A 2014 review of a series of small randomised studies found WBC offers improvements in subjective recovery and muscle soreness following metabolic or mechanical overload, but this did not seem to translate towards functional recovery [44]. A more recent review of 16 studies reported that muscle pain was reduced in 80% of studies investigating the use of WBC immediately after damaging exercise [45]. Additional benefits of WBC reported in this review included reduced inflammation, lower markers of muscle damage in the blood, and improved recovery of athletic capacity and performance (e.g., maximal voluntary contraction force) [45]. However, it is worth considering that cold water immersion has been shown to offer comparable physiological and performance recovery benefits to WBC [46,47], and may provide a more practical and less expensive alternative, which may factor into athlete/coach choice.

#### 4.1.5. Hydrotherapy (Cold Water Immersion and Contrast Water Therapy)

Hydrotherapy refers to the use of water immersion as a recovery strategy, usually via cold water immersion (CWI) or contrast water therapy (CWT—alternating between hot and cold water immersion). CWI and CWT are two of the most commonly researched and utilised recovery strategies, both by recreational and elite athletes alike. The general recommendations for temperature and duration for CWI are 11–15 °C (50–59 °F) for 11–15 min, usually in the form of a bath or portable pool [48]. For CWT, total immersion times should not be less than 10 min, with similar immersion times for both hot (38–40 °C, 100–104 °F) and cold (~10 °C, 50 °F) [49]. In general, meta-analyses and reviews on the topic tend to support CWI/CWT use as an *acute* post-exercise recovery strategy, with performance benefits for a variety of sports [48,49,50]. However, emerging research has shown that *chronic* ice-bath use following resistance training may disrupt the post-training inflammation process and blunt physiological signals and adaptations over time [51]. Although, this has not been reported following aerobic exercise performance in the chronic setting [52], whereby improvements in acute recovery may allow athletes to maintain or increase training load and thereby stimulate greater physiological adaptation [50]. Further research is required on the chronic use of cold water immersion in various sports and exercise settings, but with the available evidence at present, it may be a worthwhile strategy for certain times of the season (e.g., in-season) or during competition events (e.g., tournaments, major Games events).

#### 4.1.6. Photobiomodulation

In contrast to the previously discussed recovery strategies, photobiomodulation (PBMT) is a recovery strategy that has received less attention in the professional athletic setting, despite numerous studies on the topic in the past 10 years. PBMT is a form of low-level, non-invasive light-emitting diode or laser therapy that proposes to modulate the biological processes of cells at the mitochondrial level, increasing oxygen consumption and the production of adenosine triphosphate (ATP) [53]. Meta-analyses on the topic have generally reported positive findings for PBMT, especially in relation to reducing muscle fatigue and increases in both muscular strength and endurance performance [54,55,56]. Further, the most recent meta-analysis suggested that PBMT might be more effective than various methods of cryotherapy (cold water immersion or ice-pack application) [57]. Conversely, it is worth noting that there is a high risk of bias and a small sample size within PBMT studies [54], with many of the studies coming from the same research group. However, at this stage, PBMT does seem to have sufficient support as a recovery strategy for athletes and is largely accessible and relatively affordable.

#### 4.1.7. Active Recovery

Active recovery is a commonly used recovery modality amongst endurance and team sport athletes and usually consists of light to moderate exercise considerably below the lactate threshold [58]. Active recovery is proposed to aid muscular recovery by enhancing the clearance of blood lactate and reducing perceived muscle soreness and tenderness, particularly when compared to passive recovery [59]. A recent review of 17 studies that utilised various active recovery protocols (including yoga, cycling, and water exercise) affirmed the benefits of active recovery on soreness perception following strenuous exercise, though mixed findings were reported for performance and functional capacity indicators [60]. The intensity of active recovery should be considered, as the activity must be sufficient to raise heart rate and increase blood flow to facilitate the removal of metabolic by-products without inducing further muscle damage or increasing perceived fatigue [61]. Unlike most other recovery strategies, active recovery increases total energy expenditure and should therefore be factored into nutrition planning to maintain energy availability as well as the overall stress–recovery balance of the periodised training plan [62].

#### 4.1.8. Stretching

Post-exercise stretching is widely implemented as part of general athletic cool-downs and can include active and dynamic stretching as well as proprioceptive neuromuscular facilitation (PNF) methods. The most recent systematic review and meta-analysis on this topic analysed 10 randomised controlled trials with mostly male, recreationally trained participants [63]. It was found that post-exercise stretching was not more effective than other recovery methods (primarily passive recovery/rest) in the recovery of strength and reduction of DOMS for both short (<1 h) and longer-term (up to 72 h) periods following exercise [63]. However, if the focus of stretching is to improve range of motion, this has greater support from literature, albeit as part of a pre-exercise routine rather than recovery [64]. On the whole, research in this field tends to have poor external validity and may not reflect real-life settings where stretching is implemented, necessitating further high-quality research [63].

### 4.2. Lower Level of Evidence

The following section will discuss other recovery devices/strategies referred to in Figure 1, which currently lack a high level of research-grade evidence (i.e., meta-analysis or systematic review). However, many of these devices are popular and widely used by athletic populations, which indicates further research may be required.

#### 4.2.1. Sauna

Sauna bathing is a form of passive heat exposure using a room heated to ~70–100 °C and 15–30% relative humidity, usually for a period of 5–20 min [65]. While previous systematic and literature reviews have considered the effect of sauna bathing on cardiovascular and health parameters (e.g., blood pressure and immune function) [65,66,67], to date we are unaware of any systematic reviews or meta-analyses considering the effect of sauna use on athletic recovery following exercise. Sauna bathing following exercise may be used to promote relaxation, increase blood perfusion to the muscle, and improve injury rehabilitation [68,69]. However, the practice also imposes additional physiological stress on athletes (e.g., increased core temperature, heart rate, and sweat response) and may therefore hinder acute performance recovery, as has been shown with swimmers [70]. Meanwhile, the use of infrared saunas—which radiate heat at a much lower ambient temperature—has been shown to improve the recovery of neuromuscular function (i.e., countermovement jump height) after maximal endurance exercise [68] and resistance exercise [71]. In the absence of further evidence, post-exercise sauna bathing should be used with caution, considering potential implications for acute performance [70], unless heat acclimation is desired for a particular purpose/event.

#### 4.2.2. Recovery Boots/Sleeves

Also known as intermittent pneumatic compression or dynamic compression, these devices involve inflatable leg or arm sleeves that exert high levels of pressure and inflate and deflate in a “massage-like” action. While a plethora of studies have come out in the last 5 years on this technique, there have yet to be any systematic reviews or meta-analyses. Similar to compression garments, the majority of studies on recovery boots or sleeves report acute benefits to perceived muscle soreness, fatigue, and recovery, with mixed findings on the actual benefit to athletic performance [72,73,74]. As such, based on the current level of evidence, it may be more feasible and cost-effective to use other recovery strategies (e.g., compression garments) for similar recovery outcomes rather than recovery boots/sleeves. However, if these devices are available to the athlete, we can be confident that they are unlikely to do any harm and may provide modest recovery benefits.

#### 4.2.3. Occlusion Cuffs/Blood Flow Restriction

Similar in mechanism to recovery boots/sleeves, vascular occlusion cuffs are portable, inflatable cuffs that provide short periods of blood flow restriction (BFR) that are interspersed with recovery (reperfusion) periods for the targeted limbs [75]. The pressure exerted by such cuffs is considerably higher than that exerted by recovery boots/sleeves and compression garments in order to induce an ischemic state and improve oxygen delivery, blood flow, and muscle function with cycles of reperfusion [76]. Mixed results have been reported for the effectiveness of occlusion cuffs on recovery and subsequent performance, with some reports of small benefits to recovery of power and sprinting performance [76] and reduced muscle soreness [77] compared to control/sham groups, and other reports finding no significant changes to perceptual or performance measures [75]. Importantly, practitioners should understand the risks associated with occlusion cuffs and other forms of blood flow restriction, as these methods are not suitable for all individuals and can cause serious injury or illness if used incorrectly [78]. Further, BFR during recovery may add to the physiological stress response following training and should be considered in relation to the overall training stimulus.

#### 4.2.4. Float Tanks

Flotation-restricted environmental stimulation therapy is a method of sensory deprivation that involves athletes lying inside a light and sound-proof tank that contains a saline solution heated to skin temperature. While it is becoming a popular method of athlete recovery, with reports of professional teams including float tanks in their locker rooms or facilities, there is limited empirical evidence to consider. The few studies in this area report promising effects of floating on perceived muscle soreness, blood markers (e.g., blood lactate), fatigue, mood state, sleep, and some performance measures (e.g., sprint performance) [79,80,81]. In particular, the use of floatation in the late afternoon/evening following training appears to enhance subsequent sleep and next-day performance [80]. While these findings are positive, it is clear more research is required in this area to inform practitioners, particularly around the optimal duration and timing of float therapy.

#### 4.2.5. Massage Guns

Massage guns, or handheld percussive massage devices, deliver vibrations at varying amplitudes and are purported to reduce perceived muscle soreness and improve lower limb mobility. While they are predominantly marketed as a recovery tool, previous research has focused on pre-exercise applications of the devices, where there may be some positive changes in flexibility and muscle strength [82]. The limited research on massage guns for recovery has reported improvements in ROM [83] and benefits for restoring muscle compliance and reducing stiffness [84] with short application periods of 2–5 min. Concerningly, two case studies have reported serious illness/injury following massage gun use, including rhabdomyolysis (a severe illness caused by the breakdown of skeletal muscle fibres into the bloodstream) and hemothorax (the collection of blood in the pleural cavity, usually from blunt force trauma) [85,86]. As well, a more recent experimental study showed the potential for a small increase in lower leg muscle soreness with the use of a massage gun for 5 min after strenuous exercise [87]. Given the lack of research and the potential risk of misuse of massage guns, we suggest a cautious approach should be taken before further research can be performed.

## 5. Psychosocial Recovery

The recovery strategies/devices discussed in this review primarily target aspects of physical recovery (e.g., muscle soreness), which may in turn improve psychological recovery (e.g., improved readiness to train). However, addressing the psychosocial aspects of recovery is perhaps equally as important as the restoration of physical capacities. Various social, environmental, and emotional factors (“non-training stressors”) can contribute to psychosocial stress and changes in mood state in athletes and lead to maladaptation to training and poorer physical performance [88,89]. As such, it is important to encourage simple, proactive psychosocial strategies that suit individual athletes’ needs, such as time with friends, debriefing, counselling, relaxation techniques, and “time out” periods from training if required [88]. Allowing for sufficient psychosocial recovery outside of sport may, in turn, enhance an athlete’s capacity to cope with the physical stress incurred by training and competition within their sport. However, this element of recovery may be overlooked in favour of addressing more tangible, physical aspects of recovery, and as a result, there is comparatively less research to support the benefits of psychosocial recovery [90]. Given the high prevalence of mental health problems in elite athletes and growing awareness of the role that psychological wellbeing plays in long-term sporting success, this may be a pertinent area for future research [91].

## 6. Placebo and Belief Effects

While changes to physiological, biomechanical, neurological, or performance markers may be limited, a consistent finding for the recovery strategies discussed in this review is their tendency to improve perceptual recovery, including measures of perceived muscle soreness, pain, and fatigue. In many cases, it is difficult to design blinded research to rule out the possibility of a placebo effect with these recovery interventions; for example, participants can correctly determine when they are wearing compression socks compared to non-compressive socks [92]. While placebo effects have historically been seen as an inconvenience that needs to be controlled for in research, emerging neurobiological theory suggests that researchers and practitioners can leverage this effect to improve athletic performance [93,94]. Similarly, “belief” or “expectancy” effects—where prior belief has an observed effect on an outcome measure—can also be leveraged to improve recovery where appropriate [95], particularly when considering that many athletes rely on their coaches as a primary source of recovery information [1]. Conversely, negative beliefs regarding a particular strategy may have a detrimental effect on performance [96,97]. As such, we would recommend that coaches and practitioners establish the beliefs of the athlete prior to selecting recovery devices and strategies.

## 7. Chronic Use of Recovery Strategies

The choice and implementation of recovery strategies across micro- and meso-cycles depends on both coach and athlete preference and desired physiological or psychological outcomes. However, very little is known about the chronic use of recovery strategies over long-term periods of athletic training and their potential effect on physiological adaptations. For example, a recent systematic review and meta-analysis of eight studies reported that regular use of cold water immersion following resistance training may attenuate adaptations in maximum strength, though endurance exercise performance was unchanged [52]. This finding encourages the selective use of cold water immersion by athletes dependent on training phase and focus, an approach termed “recovery periodisation” [52]. On the other hand, we know very little about the chronic use of other recovery modalities (e.g., compression garments, sauna, cryotherapy chambers) and whether these also have the potential to impair or enhance long-term physiological adaptations. However, while there is still more to learn in this domain, athletes should not be discouraged from using recovery strategies that ultimately allow them to maintain availability for training and, thereby, the stimulus for physiological adaptations. In Table 2, we provide some guidance on when and how to implement recovery strategies across various training phases.

## 8. Conclusions

In athletic training, recovery plays a fundamental role and should be meticulously tailored to suit individual athletes and specific sports, aiming to optimise performance. Despite sleep, nutrition, and periodisation being fundamental aspects of recovery, they are sometimes disregarded in favour of new gadgets and devices claiming to alleviate soreness, reduce fatigue, and enhance performance. However, many of these claims are made without substantial scientific evidence to support their effectiveness. It must also be acknowledged that research evidence is only one consideration, and indeed, to be ahead of the curve and gain an advantage over competitors, sometimes un-tested strategies need to be trialled. Often, coaches (and athletes) apply recovery strategies based on past experiences and observations, as well as their own instincts [98]. Additionally, prior beliefs and expectations about recovery strategies can supersede negligible recovery benefits and should be considered in the process of selecting recovery strategies. Finally, psychosocial recovery and an athlete’s psychological wellbeing should also be considered as part of recovery planning, as these aspects are equally as important as physical recovery.

## Figures and Tables

**Table 1:** Proposed effects of recovery modalities on various domains perturbed during exercise and examples of recovery modalities that purport to act upon these domains.

		Example Recovery Modalities
Physiological	Improved blood flow Reduced heart rate Increased heart rate variability Decreased blood pressure Reduced skin and core body temperatures Hormonal changes (e.g., reduced cortisol and increased growth hormone) Reduced oedema Improved removal of metabolic by-products	Active recovery; compression garments; cryotherapy chambers; electromyostimulation; hydrotherapy; occlusion cuffs; photobiomodulation; sauna
Biomechanical	Improved muscle–tendon compliance Improved limb/joint range of motion Restoration of isometric strength and peak torque Decreased active and passive stiffness Decreased tissue adhesion	Compression garments; foam rolling; stretching; massage guns; recovery boots/sleeves
Neurological	Reduced muscle tension and spasm Reduction in neuromuscular excitability Reduced pain response	Compression garments; cryotherapy chambers; electromyostimulation; float tanks; foam rolling; massage guns
Psychological	Improved mood state Reduced anxiety Reduction in feelings of fatigue Increased feelings of relaxation Decreased perceived muscle soreness	Compression garments; float tanks; hydrotherapy; occlusion cuffs; photobiomodulation; recovery boots/sleeves; sauna

**Table 2:** Considerations for how to implement recovery strategies across training phases.

General Preparation/Pre-Season	Specific Preparation	Taper/Pre-Competition	Competition/Major Event
Appropriate use of recovery strategies to maximise training adaptation and goals of general preparation/pre-season phase. May involve withholding recovery strategies (e.g., CWI) to maximise adaptation (especially following resistance training).	Recovery strategies after key sessions, particularly when subsequent sessions require high levels of skill and/or high-quality training. Recovery strategies may also be utilised to reduce fatigue and soreness in preparation for key sessions.	Recovery strategies can be utilised to minimise fatigue. This may be useful to decrease the period of time required to taper effectively. Recovery strategies may be incorporated to maintain high-intensity training during this period.	Recovery strategies provided to minimise fatigue and maximise competition performance during a season or at a tournament/major event. Recovery strategies may help to manage fatigue around travel and jetlag.
